# Oral health status of patients infected with human immunodeficiency virus and related factors, Iran: a cross-sectional study

**DOI:** 10.1186/s12903-021-02002-3

**Published:** 2021-12-18

**Authors:** Soheila Shaghaghian, Mojtaba Homayooni, Maryam Amin, Fahimeh Rezazadeh

**Affiliations:** 1 Communicable Diseases Unit, Shohada-e-Enghelab Health Center, Shiraz University of Medical Science, Sibooye Boulevard, P. O. Box: 7164788363, Shiraz, Iran; 2 Shiraz HIV/AIDS Research Center, Institute of Research, Shiraz University of Medical Science, Shiraz, Iran; 3 Voluntary Counseling and Testing Center, Shiraz University of Medical Science, Shiraz, Iran; 4 School of Dentistry, Faculty of Medicine and Dentistry, University of Alberta, Edmonton, Canada; 5 Oral and Dental Disease Research Center, Department of Oral and Maxillofacial Medicine, School of Dentistry, Shiraz University of Medical Sciences, Shiraz, Iran

**Keywords:** HIV, Oral health, Dental plaque index, Periodontal index, Risk factors

## Abstract

**Background:**

Human immunodeficiency virus (HIV) continues to be a major global issue. HIV-infected patients commonly experience oral health problems. This study aimed to assess oral health status of the patients and its related factors in Shiraz, Iran.

**Methods:**

In this cross-sectional study, by using simple random sampling, 250 HIV-infected patients were selected from Shiraz Voluntary Counseling and Testing Center in 2019. Patients' decayed, missing, and filled tooth (DMFT) index, plaque index (PI), and community periodontal index (CPI) were determined. Associations between patients' characteristics and the above-mentioned indices were examined by using Pearson correlation, one-way ANOVA, chi-square, and independent sample T test. The effect of possible confounding factors was controlled by using multiple linear regression and logistic models.

**Results:**

Of the 222 participants, 111 (50%) had 15 or more missing teeth and 79 (35.6%) were edentulous. Their mean (SD) DMFT and PI were 20.71 (10.74) and 1.11 (0.79), respectively. About 40% of them had healthy gingiva. DMFT (*p* < 0.001), PI (*p* < 0.001), and CPI (*p* = 0.002) were significantly worse in men than women. The patients' DMFT and edentulous status were significantly associated with their age (both *p* < 0.001) and duration of disease (*p* = 0.001 and *p* = 0.008, respectively). Unemployed patients had the worst DMFT, PI, and CPI (all *p* < 0.001) and the highest percentage of edentulous individuals (*p* = 0.003). All examined indices were significantly worse in cigarette smokers, alcoholics, addicts, and patients with a previous history of imprisonment (all *p* < 0.001). The odds ratio of being edentulous was 5.74 times in addicted patients than in non-addicted ones (*p* < 0.001). The odds increased 0.11 with every year that the patients' age increased (*p* < 0.001). Multiple linear regression models also showed that the addicted patients had significantly more scores in DMFT index, PI, and CPI (all *p* < 0.001).

**Conclusions:**

Overall, oral health status of the HIV-infected patients was unsatisfactory. Therefore, effective interventional programs are needed for prevention and early treatment of dental problems among this population, especially for more vulnerable groups such as older men with low socioeconomic status, and those with high-risk behaviors.

## Introduction

Human immunodeficiency virus (HIV) is a global public health problem [1]. It was estimated that in 2019, 38 million people were living with HIV and 1.7 million were newly infected [2]. In Iran, similar to many other countries, the prevalence of HIV infection has rapidly increased in recent years [3]. According to the Iranian Ministry of Health and Medical Education reports, 22,406 HIV-infected patients were living in Iran in 2019 [4]. Yet, the United Nations on HIV and acquired immunodeficiency syndrome (AIDS) estimated the actual number of HIV-infected patients living in Iran in 2019 as 59,000 [5]. The introduction of antiretroviral drugs significantly reduced the mortality and increased life expectancy in HIV-infected patients [6]. Increasing the life expectancy required more attention to the management of the problems that these patients experience such as oral health problems, which was one of the earliest signs of HIV infection occurring in 30–80% of HIV-infected patients [7].

Several studies showed high prevalence of periodontal disease, tooth loss, and tooth decay in HIV-infected patients [8–14]. In Brazil, the mean of decayed, missing, and filled teeth (DMFT) index in the patients was 12.4 and only 11.6% of them did not have periodontal disease [8]. About 41.5% and 62% of the patients needed upper and lower denture, respectively [9], and 72.4% needed some form of dental treatments, mainly fillings [10]. In Thailand, oral problems were reported in 84.3% of the patients [11] and oral candidiasis in 65% of them [12]. In India, nearly all the patients needed one form of dental treatment and only 12% of them had healthy periodontium [13]. Only 5.8% of children living with HIV in Cambodia were free from caries and the mean number of their decayed teeth was 7.7 [14].

Similarly, a study conducted in Iran showed poor oral health status of HIV-infected patients [3]. In the study conducted in Tehran, capital of Iran, the mean DMFT in HIV-infected patients was 19.8 [3], which was significantly higher than that of general population [15]. The HIV-infected patients also had a high prevalence of periodontal disease so that only 1.5% of them had a healthy periodontium. Furthermore, 31% of the participants had at least one recognized mucosal-oral lesion related to HIV/AIDS and 15.9% of them were edentulous [3].

While immune system suppression has been recognized as the main cause of oral problems in HIV-infected patients [16], lack of attention to oral health can also affect the frequency of the problems in these patients. One of the main reasons of poor oral health in HIV-infected patients is their low-use of dental services [9, 17]. In a study conducted in the United States of America, while 48.2% of patients reported an unmet oral health need since diagnosing as HIV-positive, the majority (52.4%) had not seen a dentist in more than two years [17]. In another study, HIV-infected patients declared that their low-use of dental services was because of the high-cost of the services and their fear of rejection by dentists [3]. Poor oral health leads to tooth loss in many patients and aggravates their poor nutritional status. Therefore, routine dental care can significantly improve their quality of life [8]. However, fear of contracting the virus leads some dentists to refuse to provide services to these patients [18].

Because of significant associations found between patients' unmet dental needs and oral health [3], health authorities have established public centers for management of HIV-infected patients’ oral health in Iran. The centers provide very limited services, mainly for treatment of dental caries, and do not offer any preventive cares. To establish comprehensive programs for promoting oral health in HIV-infected patients, policymakers should be aware of the prevalence of oral health problems and their impact in this population. Therefore, the aims of this study were to evaluate the oral health status of HIV-infected patients and the related factors in Shiraz, Iran.

## Method

### Participants

This cross-sectional study conducted in the Voluntary Counseling and Testing (VCT) center affiliated to Shiraz University of Medical Sciences in 2019. All HIV-infected patients residing in Shiraz, a city in the south of Iran, should be referred to this center. In the center, they received antiviral medication and other necessary prophylactic and therapeutic measures. The target population of the present study was the HIV-infected patients who were followed up by the center. We excluded patients in prisons and those without a valid telephone number from the study because of their unavailability. We also excluded patients aged less than eighteen years and those who did not give consent or were uncooperative.

### Sample size calculation and sampling

We calculated sample size by using the plaque index (PI), community periodontal index (CPI) and DMFT index determined in previous studies [3, 8], α = 0.05, and d = 0.1. The sample size was corrected for the finite population and determined as 179 patients. We increased the sample size to 250 to compensate the possible withdrawal of patients from the study. Using national software for control of HIV/AIDS in Iran, we made a list including the code number of all HIV-infected patients who were under follow-up by Shiraz VCT center. Using SPSS software, we selected 250 of the codes by simple random sampling.

### Data collection

One of the faculty members of Shiraz Dental School first trained the dentist who collected the data; they both then separately performed oral examinations for 18 patients and determined the DMFT index, PI, and CPI for each patient. No significant differences were found between the two set of measures using Wilcoxon signed ranks test.

A VCT center staff also helped us in collecting data. She obtained informed consent from the selected patients after giving adequate information about the study and its objectives. She made an appointment for the consented patients at the dental clinic dedicated to the HIV-infected patients. To enhance patients' engagement in the study, some incentives were offered including free dental services with a minimum waiting time, transportation cost, and an honorarium to compensate their time off work.

Patients' demographic characteristics such as age, sex, marital status, number of their children, education, employment, and history of imprisonment were recorded. The VCT staff also collected information about patients' general and oral health such as duration of disease, route of HIV transmission, smoking cigarette/hookah, drinking alcohol, addiction to smoked /injected illegal drugs either through interviews with the patients or reviewing their charts in the VCT center. The dentist examined all patients' teeth and gingiva using a dental unit and determined their edentulous status, DMFT index, PI, and CPI.

DMFT index was determined according to World Health Organization (WHO) criteria by using disposable plane mouth mirrors and probes. The results were recorded in oral health assessment forms designed by WHO in 2013 [19].

To evaluate dental cleanness, the dentist determined the PI introduced by Loe and Silness by using disposable plane mouth mirrors and dental explorers [20]. He examined four gingival areas, i.e. distobuccal, buccal, mesiobuccul, and lingual of the teeth numbered 16, 12, 24, 36, 32, and 44. If one of the mentioned teeth was not present, he examined all the teeth. First, he dried the examined tooth by using a cotton roll and placed it on the vestibule above the tooth to prevent getting wet again. Then, he determined the score of each gingival area of the tooth based on the presence of plaque on the cervical third of the tooth as below:

- Score 0: No plaque
- Score 1: A layer of plaque adhered to the free margin of gingiva and adjacent area of the tooth that could be seen only by probing
- Score 2: Moderate accumulation of soft deposits within the gingival pocket, or on the tooth or gingival margin that could be seen with the naked eye
- Score 3: Large amount of soft material within the gingival pocket, or on the tooth or gingival margin

The mean of the scores given to the four gingival area of the examined tooth was considered as the PI of the tooth. The PI of each patient was determined by calculating the mean of the PI of all examined teeth.

CPI was used to evaluate patients' gingival health status. The dentist determined the index after probing the teeth numbered 11, 16, 17, 26, 27, 31, 36, 37, 46, and 47. He used a WHO probe to measure the depth of gingival sulcus of the index tooth. If one of the teeth was not present, he examined the depth of gingival sulcus of all teeth presenting in that sextant of the mouth. After probing, he scored the gingiva of each index tooth as below:

- Score 0: Healthy gingiva
- Score 1: Gingival bleeding
- Score 2: Calculus and gingival bleeding
- Score 3: Shallow periodontal pockets (4 to 5 mm)
- Score 4: Deep periodontal pockets (6 mm or more)

The patients’ CPI was determined as the highest score given to their gingiva [21].

### Statistical methods

Using SPSS software version 18 (SPSS Inc., Chicago, Illinois, USA), we analyzed the collected data. The prevalence of the patients' edentulous status, and mean and standard deviation of their DMFT index, PI, and CPI were determined. Using Pearson correlation, one-way ANOVA, chi-square, and independent sample T test, we evaluated the association between patients' characteristics and the recorded indices.

To control the effect of confounding factors on edentulous status as dependent variable, we used logistic regression analysis with forward: Wald's method. In addition, multiple linear regression models with enter method were used to adjust the effect of confounding factors on the DMFT index, PI, and CPI as dependent variables. To control multicollinearity, we used factor analysis. According to the result of the analysis, the independent variables, which were analyzed in unadjusted models, divided into 3 groups:

- Group1: sex, history of imprisonment, smoking cigarette/hookah, drinking alcohol, addiction to smoked illegal drugs, addiction to injected illegal drugs, route of HIV transmission, employment
- Group 2: age, duration of disease, number of children, education
- Group 3: marital status

The variables in group 1 had strong correlations with each other; therefore, we selected “addiction to smoked illegal drugs” from this group. Similarly, since strong correlations were found among the variables in group 2, we selected “age” as representative of this group. As a result, only three variables of “addiction to smoked illegal drugs”, “age”, and “marital status” were included in the logistic and multiple linear regression models. An alpha level of 0.05 was regarded as statistical significance.

### Ethical considerations

All stages of the investigation were conducted in accordance with the principles of the Helsinki Declaration. The Research Ethics Committee of Shiraz University of Medical Sciences reviewed and approved the study protocol (≠ IR.SUMS.REC.1394.S863, the date of ethical approval: 16 November 2018). Ethical considerations were considered during each step of the research process. Informed consent was obtained from all patients. The patients' identity was kept anonymous for all members of the research team as the information was recorded based on their code in the HIV/AIDS national software.

## Result

### Participants

Of the 250 selected patients, 222 (88.8%) participated in the study. Thirteen selected patients did not consent to participate in the study. Three under 18-year-old and seven uncooperative patients were excluded from the study. Five patients were also excluded because no telephone number was recorded for them in the VCT center.

### Descriptive data

Of 222 participants, 151 (68%) were men. The age of the participants ranged from 22 to 73 years with a mean (SD) of 39.7 (8.6). They were diagnosed for HIV-infection between 1996 and 2019; therefore, the duration of their disease ranged from 2 to 25 years with the mean (SD) of 7.61 (4.21) years. Eighty-nine (40.64%) of them had less than 5 years education and 118 (53.88%) were unemployed or had a temporary job (Table 1).

**Table 1:** Participants' demographic and health characteristics and relationship between the characteristics and their edentulous status (N = 222)

Variables	Measures	Univariate analysis		
		Participants' edentulous status		*p*-value
		Yes	No	
Age, year, Mean ± SD	39.68 ± 8.62	43.61 ± 8.60	37.51 ± 7.84	0.001^¶^
Sex, N (%)				0.001^¶¶^
Man	151 (68.02)	65 (43.05)	86 (56.95)	
Woman	71 (31.98)	14 (19.72)	57 (80.28)	
Duration of disease, year, Mean ± SD	7.61 ± 4.21	8.70 ± 4.78	7.07 ± 4.09	0.008^¶^
Marital status, N (%)				0.294^¶¶^
Single	77 (34.68)	32 (41.56)	45 (58.44)	
Divorced/widowed	61 (27.48)	22 (36.06)	39 (63.94)	
Married	84 (37.84)	25 (29.76)	59 (70.24)	
Number of their children, Mean ± SD	0.97 ± 1.39	1.01 ± 1.62	0.94 ± 1.25	0.726^¶^
Education, N (%)				0.277^¶¶^
0–5 year education	91 (40.99)	38 (41.76)	53 (58.24)	
6–11 year education	89 (40.09)	28 (31.46)	61 (68.54)	
Having a diploma degree or university education	42 (18.92)	13 (30.95)	29 (69.05)	
Employment status, N (%)				0.003^¶¶^
Unemployed or having a temporary job	118 (53.15)	52 (44.07)	66 (55.93)	
Having a permanent job	36 (16.22)	14 (38.89)	22 (61.11)	
Homemaker	68 (30.63)	13 (19.12)	55 (80.88)	
Route of HIV transmission, N (%)				0.006^¶¶^
Using share syringe by IV-drug abusers	128 (57.66)	58 (45.31)	70 (54.69)	
Sexual contact outside the family	46 (20.72)	10 (21.74)	36 (78.26)	
Transmission from her/his HIV^ +^ spouse	37 (16.67)	9 (24.32)	28 (75.68)	
Other routes	11 (4.95)	2 (18.18)	9 (81.82)	
Smoking cigarette/hookah (up to now), N (%)				< 0.001^¶¶^
Yes	150 (67.57)	68 (45.34)	82 (54.66)	
No	72 (32.43)	11 (15.28)	61 (84.72)	
Drinking alcohol (up to now), N (%)				0.001^¶¶^
Yes	96 (43.24)	46 (47.92)	50 (52.08)	
No	126 (56.76)	33 (26.19)	93 (73.81)	
Addiction to smoked illegal drugs (up to now), N (%)				< 0.001^¶¶^
Yes	138 (62.16)	66 (47.83)	72 (52.17)	
No	84 (37.84)	13 (15.48)	71 (84.52)	
Addiction to injected illegal drugs (up to now), N (%)				< 0.001^¶¶^
Yes	119 (53.60)	59 (49.58)	60 (50.42)	
No	103 (46.40)	20 (19.42)	83 (80.58)	
History of imprisonment, N (%)				< 0.001^¶¶^
Yes	130 (58.56)	62 (47.69)	68 (52.31)	
No	92 (41.44)	17 (18.48)	75 (81.52)	

*HIV* human immunodeficiency virus; *IV* intravenous; *SD* standard deviationl

^¶^Indepandent sample *T* test

^¶¶^Chi-square test

### Calibration

Calibration of the data collector was assured by comparing his measures of DMFT, PI, and CPI with those of a faculty member of Shiraz dental school in 18 patients. The median (range) of DMFT index, PI, and CPI measured by the dentist and faculty member were [14 (4–24) vs. 14 (3–25), *p* = 0.783], [0.54 (0.03–1.70) vs. 0.52 (0.03–1.25), *p* = 0.400], and [2 (0–4) vs. 2 (0–4), *p* = 0.564], respectively. No significant differences were found between the indices measured.

### Participants' oral health status

Of all participants, 79 (35.6%) were edentulous and 143 (64.4%) had at least one tooth. The mean (SD) number of existed teeth in non-edentulous patients was 23.13 (7.45). Of edentulous patients, only 24 (30.4%) wore complete denture. In addition, 7 patients who had at least one tooth wore a partial denture. Of 143 participants with at least one tooth, only 65 (45.5%) reported brushing their teeth daily. However, 10 (7.0%), 30 (21.0%), and 11 (7.7%) had brushed two-six times per week, once per week, and once per month, respectively. Nevertheless, 27 (18.9%) never brushed their teeth. While 92 (64.3%) used toothpaste all the time, 6 (4.2%), 5 (3.5%), 6 (4.2%) used toothpaste most of the time, sometimes, and rarely, respectively, and 34 (23.8%) never used toothpaste.

Of the participants, 127 (57.2%) did not have any decay and 168 (75.7%) did not have any fillings. The mean (SD) number of decayed and filled teeth were 2.71 (4.60) and 2.06 (0.90), respectively. The number of missing teeth was much more than the number of filled and decayed teeth. While only 6.8% of participants had all their teeth, 50% of them had 15 or more missing teeth. The mean (SD) number of missing teeth was 17.10 (12.61). The DMFT indices varied from 0 to 32 (median = 21). The indices were 32 in 85 participants and 0 only in 3 participants with a mean (SD) of 20.71 (10.74).

The PI of 143 participants that had at least one tooth varied from 0 to 3. While 50% of them had PI ≤ 1 (median = 1), only 21 (9.5%) of them were determined to have PI = 0. The Mean of their PI was 1.11 ± 0.79.

We evaluated participants' gingiva using CPI. Of the participants, 57 (39.9%), 39 (27.3%), and 36 (25.2%) had scores of 0, 1, and 2, respectively. Only 8 (5.6%) participants had a score of 3 and 3 (2.1%) participants had a score of 4.

### Relationship between participants' characteristics and their edentulous status

The participants edentulous status was not associated to their education (*p* = 0.277), number of children (*p* = 0.726), and marital status (*p* = 0.294). Nevertheless, the edentulous status was more prevalent in men than in women (*p* = 0.001) and the prevalence was increased by increasing age (*p* = 0.001) and duration of disease (*p* = 0.008). It was also more in those had ever smoked cigarette/hookah (*p* < 0.001), drunk alcohol (*p* = 0.001), and addicted to smoked/injected illegal drugs (both *p* < 0.001). Comparing those infected with HIV by various routes, we found that the prevalence was the most in intravenous (IV)-drug abusers using share syringe (*p* = 0.010, Table 1).

### Logistic regression model

The goodness of fit in logistic regression model was checked by Hosmer and Lemeshow Test (*p* = 0.446). The model excluded the variable of marital status from the final step of the analysis. However, it showed significant associations between the edentulous status and the two other independent variables (i.e. age and addiction to smoked illegal drugs).The odds ratio of being edentulous was 5.74 times in addicted patients than in non-addicted ones. The odds ratio was 1.11 for age, i.e. the odds of being edentulous in HIV-infected patients increased 0.11 with every year increase of their age (Table 2).

**Table 2:** Logistic regression models with participants' edentulous status as dependent variables (N = 222)

Participants’ characteristics	Logistic regression		
	OR	95%CI of OR	*p*-value
Marital status	–	–	–
Addiction to smoked illegal drugs	5.74	2.70–12.18	< 0.001
Age	1.11	1.06–1.16	< 0.001

### Relationship between participants' characteristics and their oral heath indices

Most factors related to participants' edentulous status were related to the indices, as well. The means of DMFT index (*p* < 0.001), PI (*p* < 0.001), and CPI (*p* = 0.002) were higher in men than women. They were significantly higher in those who smoked cigarette/hookah, drank alcohol, and addicted to smoked/injected illegal drugs. Among those infected with HIV by various routes, the means were the highest in IV-drug abusers using share syringe. With increasing the participants' age (*p* < 0.001) and duration of disease (*p* = 0.001), their DMFT indices increased but the two variables did not significantly associate to PI and CPI (Tables 3 and 4).

**Table 3:** Relationship between the participants' qualitative characteristics and their oral heath indices (N = 222)

Participants' characteristics	DMFT Index		Plaque Index		Community Periodontal Index	
	Mean ± SD	*p*-value	Mean ± SD	*p*-value	Mean ± SD	*p*-value
Sex		< 0.001^¶^		< 0.001^¶^		0.002^¶^
Man	23.32 ± 9.85		1.35 ± 0.79		1.23 ± 1.07	
Woman	15.15 ± 10.51		0.75 ± 0.65		0.72 ± 0.90	
Marital status*		0.003^¶¶^		0.001^¶¶^		0.340^¶¶^
Single	23.52 ± 10.03^a^		1.42 ± 0.75^a^		1.20 ± 0.99	
Divorced/widowed	21.21 ± 10.38^ab^		1.15 ± 0.82^ab^		1.02 ± 1.01	
Married	17.76 ± 10.99^b^		0.85 ± 0.72^b^		0.90 ± 1.08	
Education*		0.001^¶¶^		0.074^¶¶^		0.992^¶¶^
0–5 year	23.16 ± 9.97^a^		1.22 ± 0.79		1.06 ± 0.86	
6–11 year	20.32 ± 9.97^ab^		1.17 ± 0.74		1.03 ± 1.14	
Having a diploma degree or university education	15.67 ± 12.04^b^		0.81 ± 0.84		1.03 ± 1.12	
Employment status ^*^		< 0.001^¶¶^		< 0.001^¶¶^		< 0.001^¶¶^
Unemployed or having a temporary job	23.67 ± 9.67^a^		1.49 ± 0.71^a^		1.44 ± 1.04^a^	
Having a permanent job	20.67 ± 11.13^a^		0.90 ± 0.83^b^		0.54 ± 0.86^b^	
Homemaker	15.43 ± 10.38^b^		0.73 ± 0.65^b^		0.73 ± 0.91^b^	
Route of HIV transmission^*^		< 0.001^¶¶^		< 0.001^¶¶^		0.020^¶¶^
Using share syringe by IV-drug abusers	24.08 ± 9.42^a^		1.39 ± 0.79^a^		1.27 ± 1.07^a^	
Sexual contact outside the family	16.48 ± 11.18^b^		0.93 ± 0.72^b^		0.89 ± 1.06^ab^	
Transmission from her/his HIV^+^ spouse	16.35 ± 10.42^b^		0.86 ± 0.66^b^		0.82 ± 0.82^ab^	
Other routs	13.82 ± 10.92^b^		0.47 ± 0.63^b^		0.34 ± 0.71^b^	
Smoking cigarette/hookah (up to now)		< 0.001^¶^		< 0.001^¶^		< 0.001^¶^
Yes	23.91 ± 9.52		1.39 ± 0.78		1.34 ± 1.08	
No	14.04 ± 10.11		0.73 ± 0.64		0.61 ± 0.80	
Drinking alcohol (up to now)		0.001^¶^		0.017^¶^		0.001^¶^
Yes	23.36 ± 10.13		1.32 ± 0.76		1.42 ± 1.11	
No	18.68 ± 10.79		0.99 ± 0.78		0.82 ± 0.93	
Addiction to smoked illegal drugs (up to now)		< 0.001^¶^		< 0.001^¶^		< 0.001^¶^
Yes	24.14 ± 9.63		1.43 ± 0.79		1.36 ± 1.06	
No	15.06 ± 10.11		0.79 ± 0.66		0.69 ± 0.89	
Addiction to injected illegal drugs (up to now)		< 0.001^¶^		< 0.001^¶^		< 0.001^¶^
Yes	24.76 ± 9.39		1.48 ± 0.81		1.42 ± 1.11	
No	16.03 ± 10.33		0.85 ± 0.66		0.75 ± 0.88	
History of imprisonment,		< 0.001^¶^		< 0.001^¶^		0.001^¶^
Yes	24.43 ± 9.39		1.42 ± 0.76		1.32 ± 1.10	
No	15.44 ± 10.36		0.83 ± 0.71		0.76 ± 0.90	

*DMFT* decayed, missing, and filled teeth; *HIV* human immunodeficiency virus; *IV* intravenous

^¶^Indepandent sample T test

^¶¶^One way ANOVA

^*^Different letters show statistically significant differences

**Table 4:** Relationship between the participants' quantitative characteristics and their oral heath indices (N = 222)

Participants' characteristics	DMFT Index		Plaque Index		Community Periodontal Index	
	r	*p*-value	r	*p*-value	r	*p*-value
Age	0.389	< 0.001	0.083	0.326	− 0.008	0.926
Duration of disease	0.231	0.001	0.069	0.415	0.149	0.075
Number of children	0.004	0.957	− 0.178	0.034	− 0.151	0.072

*DMFT* decayed, missing, and filled teeth

*r* Pearson Correlation

### Multiple linear regression models

The goodness of fit in multiple linear regression models were checked by using adjusted R square. For the models with DMFT index, PI, and CPI as dependent variables, the adjusted R squares were 0.282, 0.179, and 0.100, respectively. The models showed that considering the confounding factors, we found significantly higher scores of DMFT index, PI, and CPI in the patients addicted to smoked illegal drugs than the non-addicted ones (all *p* < 0.001). Furthermore, single patients, compared to married ones, had a significantly higher DMFT index (*p* = 0.008) and PI (*p* = 0.013). The patients' age was also significantly associated with DMFT index (*p* < 0.001); with every year increase in patients' age, DMFT index increased by 0.456 (Table 5).

**Table 5:** Multiple linear regression models with participants' oral heath indices (DMFT index, plaque index, and community periodontal index) as dependent variables (N = 222)

Participants' characteristics	DMFT Index		Plaque index		Community Periodontal Index	
	B	*p*-value	B	*p*-value	B	*p*-value
Marital status						
Single	4.077	0.008	0.381	0.013	0.031	0.885
Divorced/widowed	2.353	0.124	0.276	0.064	0.111	0.591
Married	Reference Group		Reference Group		Reference Group	
Addiction to smoked illegal drugs	7.135	< 0.001	0.539	< 0.001	0.679	< 0.001
Age	0.456	< 0.001	0.007	0.359	− 0.005	0.645

*DMFT* decayed, missing, and filled teeth

*B* regression coefficient

## Discussion

This cross-sectional study evaluated the oral health status of HIV-infected patients living in Shiraz and factors affecting their status. Although half of the patients had 15 or more missing teeth and about one third of them were edentulous, among them, only about 14% were wearing partial or complete denture. The DMFT score of 50% of participants was more than 21 and all oral heath indices were worse in men, older patients, and those with longer duration of HIV infection. The patients with high risk behaviors such as smokers, alcoholics, addicts, and those with a history of imprisonment had poorer oral health status.

The high DMFT score found in the present study was very similar to that of another Iranian study (DMFT = 19.8) [3]. The higher DMFT score in HIV-infected patients as compared with that in Iranian general population (DMFT = 7.33) [15] highlighted the severity of dental problems in this group. The mean DMFT score in HIV-infected patients was similarly higher than that in non-infected ones in an Indian study, as well [22]. In the present study, similar to another Iranian study [3], the number of missing teeth (mean = 17.10) was more than the number of filled (mean = 2.06) and decayed (mean = 2.71) teeth. The high number of missing teeth in the present study as compared to others [3, 8, 13, 23] might be because of the number of older patients. Furthermore, extraction was reported as most frequent service that HIV-infected patients received because these patients tended to attend emergency dental visits where mainly led to tooth extraction instead of restorative treatments [3]. Another reason might be the poor oral hygiene among these patients. In the present study, only 45.5% of the dentate patients declared that they brushed their teeth daily. Therefore, to improve the situation, we recommend policymakers to develop and implement educational programs to promote tooth brushing and other preventive modalities among this population. Furthermore, the high number of missing teeth might be because of patients’ socioeconomic status, which forced them to decide to extract treatable teeth. Therefore, educational programs are recommended to highlight the importance of preserving teeth and potential consequences of losing natural teeth. In addition, because of patients’ socioeconomic status and their fear of rejection by dental providers, dental clinics dedicated to HIV-infected patients, where the patients can be treated at a subsidized cost are needed. A dental clinic in Shiraz and similar clinics in other large cities in Iran are dedicated to HIV-infected patients. The high score of DMFT index and low percentage of patients wearing denture in the present study indicate that the provided services are inadequate or there may be some other barriers preventing these patients from receiving the offered services. Further qualitative research is warranted to explore these barriers from patient’s perspective.

Patients’ PI in the present study (PI = 1.11) was very similar to that in Indian studies with the same population [PI = 1.17 [24] & 1.40 [25]] but a South African study reported a much higher PI [PI = 2.55 [26]]. Using CPI, we found healthy gingiva in about 40% of the patients, but in another Iranian study, only 1.5% of the patients had a healthy periodontium [3]. An Indian study also reported healthy gingiva in 21.2% of HIV-infected patients [27] while in two other Indian studies, only 2% [13] and 0.8% [22] of the patients had healthy gingiva. The discrepancy in findings of these studies might be the result of the participants' oral hygiene because a significant association was confirmed between oral hygiene habits and periodontal status in HIV-infected patients [26]. In a study conducted in Brazil, 85% of the HIV-infected patients cleaned their teeth once a day and about 84% of them had healthy gingiva [28]. Overall, researchers found poorer periodontal status in patients with HIV as compared to non-HIV patients [22]. Periodontal problems might be the first sign of HIV infection in oral cavity because immune suppression could create the change in oral tissue and micro flora that could lead to periodontal disease. Side effects of the drugs used for the patients, poor oral hygiene, and smoking could also deteriorate the periodontal status [27]. Therefore, practical guidelines should be established for improving home care of these patients. Regular periodontal check-ups, early and appropriate treatment of periodontal disease, and smoking cessation programs should also be facilitated for them through public clinics.

All oral heath indices evaluated in the present study were worse in men than women. Another Iranian study also found that missing teeth was more prevalent in male individuals than female ones. However, after controlling the confounding factors such as smoking, the researchers could not find the association between sex and missing tooth [29]. Therefore, poor oral health indices in men might be the effects of other health behaviors such as smoking, which was more prevalent in men. In the present study, similar to other studies [8, 23], by increasing the participants' age, their DMFT indices increased. The odds of being edentulous increased 0.11 with every year that the patients' age increased. Older HIV-infected patients suffered from more comorbidities than younger ones and patients with comorbidities had higher DMFT indices [8]. Poor oral health indices in older patients could also be explained by more dental extractions due to caries, and periodontal diseases [29]. Therefore, we should consider male and older patients as vulnerable groups, and implement more intensive preventive measures for the groups.

The present study showed a significant association between some oral health indices and indicators of socioeconomic status. For example, similar to other studies [3, 9, 30], we found unemployed patients or those with a temporary job to have the worst oral health indices. Patients with permanent job had more income and more access to dental services. They might have dental insurance; therefore, they were more likely to use dental care services including dental check-ups regularly [31]. We also found a significant association between patient’s education and some of oral health indices, which is similar to other reports [9, 23]. Less educated people may experience poor oral health because of lack of knowledge about oral hygiene [32, 33]. In the present study and other studies [3, 9], oral health indices were significantly associated with patients' marital status too. The relationship between the oral health indices and socioeconomic factors emphasized on the fact that high cost of oral health care services was one of the most important factors that limited the utilization of dental services among HIV-infected patients. Providing appropriate health insurance and essential facilities for these patients is an effective intervention in promoting their oral health status.

In the present study, the patients' oral health indices were significantly associated to factors related to HIV infection including duration of HIV infection and route of HIV transmission. In the present study, similar to another study [23], oral health status was worse in patients suffering from HIV infection for a longer period. Likewise, more unmet dental needs [18] and more prosthetic need [30] were found in HIV-infected patients with more years since being diagnosed. Other studies also showed the association between poor oral health indices in the patients and other related factors of HIV infection, i.e. high viral load [34] and advanced stages of HIV infection [3]. The results indicated that better oral health was dependent on better HIV control and confirmed the association between general and oral health. Therefore, to promote the patients' oral health, policymakers should design programs for better control of their HIV infection. Moreover, comparing those infected with HIV by various routes, we found the poorest oral health indices in IV-drug abusers using share syringe. Other studies also showed a significant association between route of HIV transmission and some oral health indices such as number of missing teeth [23] and DMFT index [9]. The result highlights the importance of establishing educational and supportive programs for IV-drug abusers.

In the present study, similar to other studies [9, 29, 30], patients' oral health indices were significantly worse in those who smoked cigarette/hookah than in the non-smokers. This might be because of low PH [35] and inadequate buffering capacity of smokers' saliva [36]. Compared with non- alcoholic patients, alcoholic ones, in the present study and other studies [1, 37], had worse oral health indices. Gupta and coworkers [38] showed lower salivary flow rate and PH in alcoholics than non-alcoholics, which could be the reason for their poor oral health. Similar to Santo and coworkers' study [30], we also found worse oral health indices in patients addicted to smoked/injected illegal drugs. The odds ratio of being edentulous was 5.74 times in addicted patients than in non-addicted ones. The result was predictable because previous studies showed serious oral health problems including generalized dental caries, periodontal diseases, and tooth loss in drug abused individuals who were not infected by HIV [39]. In the present study, the oral health indices were also worse in patients with a history of imprisonment, which was similar to what Vainionpää and coworkers reported. They found poor oral health status in prisoners so that 80% of them had the need for restorative treatment. In their study, the mean DMFT score of prisoners was 17 and no one had healthy periodontium [40]. Prisoners had harmful oral health-related behaviors [40], which leaded to poor oral health [41]. They also had high level of unmet dental need because of physical, financial, or cultural barriers to dental care [41]. Their personal knowledge, attitude, and behavior regarding seeking and maintaining oral health could also aggravate the problem y(41). Therefore, we recommend policymakers to consider prisoners or persons with a history of imprisonment as a vulnerable group. To meet the unmet needs of vulnerable groups such as cigarette smokers, alcoholics, drug addicts, and prisoners, policymakers should establish health promotion programs.

Although we tried our best to conduct a well-designed study, some limitations need to be acknowledged. First, the study was cross-sectional so all limitations of this type of study should be considered. The most important was about the casual inferences between the variables, which was questionable. Therefore, to determine factors affecting oral health status of HIV-infected patients accurately, a prospective cohort study would be more appropriate. The second limitation was selection of participants from a single VCT center, which was the only referral center for HIV-infected patients in Shiraz. It was a national requirement that all HIV-infected patients were referred to a VCT center but some patients did not comply to attend the center; therefore, we did not have access to them. It is possible that patients refusing regular follow-up care experienced worse health status than those being under the care. Therefore, the oral health status of all HIV-infected patients in Shiraz might be worse than what we report. For better evaluation, a community-based study is recommended. Finally, patients with HIV generally have other comorbidities such as diabetes mellitus that can be confounding for oral health outcomes. Therefore, to evaluate factors affecting oral health status of this population accurately, other comorbidities should also be considered in the future studies.

## Conclusions

The oral health status of HIV-infected patients was not satisfactory. The mean missing and decayed teeth scores, periodontal condition, and prosthetic needs demonstrated a high level of unmet need in this population. Therefore, dental services should be provided free of charge or with subsidies to HIV-infected patients to reduce their unmet dental needs. In addition, effective collaboration between medical and dental professionals is essential for improvement of oral health outcomes since good oral health was found to be associated with proper control of the disease among patients with HIV. Poor dental cleanness and periodontal status in the patients of the present study, that confirmed by PI and CPI, and low frequency of tooth brushing highlighted the importance of preventive dental measures in any oral health policies. The measures consist of oral health education about proper tooth brushing and flossing methods, regular dental check-ups, and interventional programs for decreasing high risk behaviors. Oral health indices were worse in men, especially older ones, and those with longer duration of HIV infection and high risk behaviors such as cigarette smokers, alcoholics, and illegal drug addicts. Therefore, more intensive preventive programs are necessary for these vulnerable groups.

## Data Availability

The datasets during the current study are not publicly available due to confidentiality of the patients’ data, but they will be available upon editorial reasonable request**.**

## Abbreviations

HIV: Human immunodeficiency virus
AIDS: Acquired immunodeficiency syndrome
DMFT: Decayed, missing, and filled tooth
PI: Plaque index
CPI: Community periodontal index
VCT: Voluntary counseling and testing
WHO: World Health Organization
ANOVA: Analysis of variance
